# Post-Partum Hemorrhage

**Published:** 1880-04

**Authors:** 


### POST-PARTUM HEMORRHAGE.

In the clinical records of the Rotunda Hospitals, presented to the Obstetrical Society of Dublin, the following interesting report was made by Lombe Atthill, M. D., Master:

*Post-Partum Hemorrhage.*—There were thirty cases of *post-partum* hemorrhage, some of which were of an alarming nature. The use of hot water in the treatment of this complication was very frequently employed both in the hospital and extern maternity, and has proved eminently satisfactory. It has, indeed, much to recommend it, for not only is it a powerful hæmostatic and excitant of uterine contraction, but it is also a general stimulant. If used with ordinary care it is not only harmless, but beneficial, by thoroughly cleansing the uterus from clots, portions of membrane, &c., which may have been left in its cavity; and, what is a matter of great importance, it is always at hand when wanted. It will not, we are of opinion, be found altogether to displace the use either of cold water, or of the perchloride of iron, but rather to be applicable to a distinct class of cases, in which the former of these remedies would be unsuitable and the latter unnecessary.

Dr. Atthill was first induced to try the use of hot water in the treatment of uterine hemorrhage, in the earlier part of the time embraced in this report, in consequence of a letter written by Dr. Whitwell, of San Francisco, to Dr. Foley, of Boston, who was studying at the time in this hospital.

The method of carrying out the practice is exceedingly simple. An ordinary syphon-syringe is the only instrument required, though we now use one with a long vulcanite nozzle specially constructed for vaginal and intra-uterine injection. This is carried up to the fundus, and, with the usual precautions against injecting air, and securing a free return, we inject water as hot as can be conveniently borne by the hand—*i. e.*, about 112° F.—in a full stream into the cavity, continuing thus until a good contraction is secured, and the water returns quite clear and colorless.

The following are some of the results of our experience in the use of hot water:—

1st. In cases of sudden and violent hemorrhage in a strong and plethoric woman it is better first to use cold.

2d. Where from the prolonged or injudicious use of cold, the patient is found shivering and depressed, the beneficial effect of injecting hot water is rapid and remarkable.

3d. In nervous, depressed, and anæmic women, hot water may at once be injected, without previously using cold.

4th. In cases of abortion, where from uterine inertia the ovum, although separated from the uterine wall, is wholly or in part retained, the injection of hot water is generally followed by most satisfactory results.

5th. Where the injection of the perchloride of iron is considered necessary, previous injection of hot water clears the uterus of clots, &c., permitting the fluid to come directly in contact with the bleeding surface, and lessening the chance of septic absorption.

The following cases will illustrate its effects :—

Case I.—E. D., aged twenty-four. Labor was tedious, and completed finally with forceps; afterwards there was some sharp *post-partum* hemorrhage, which was temporarily arrested by cold and ergot. The uterine contraction was not, however, satisfactory, and more or less draining continued for some time after delivery. Hot water was at length injected, and was followed by firm and permanent contraction.

*Prolonged Use of Cold Water; Successful Injection of Hot Water.*—Case II.—M. H., aged twenty-seven; fifth confinement. This patient had had hemorrhage after former labors. On this occasion the process proceeded naturally and rapidly until after the birth of the infant; but some hemorrhage preceded the expulsion of the placenta, which was accompanied by a quantity of blood and clots. The flooding now became considerable. The pupil in charge of the case summoned the assistant physician, and in the meantime tried to check the hemorrhage with cold water, which he not only injected into the vagina and uterus, but also poured from a height upon the abdomen. The patient was found at about four o'clock, on a raw spring morning, with her bed and linen saturated with cold water, depressed and shivering, with blanched face, and small, feeble and rapid pulse—135 per min. A small stream of blood was still flowing from the vulva; the uterus being relaxed and distended, a quantity of clots were expelled by friction and pressure, and hot water immediately injected. There was no more hemorrhage, and when the bed linen had been changed and a hot jar applied to her feet, the patient expressed the greatest satisfaction; her pulse had fallen to 115. She had subsequently an attack of pleurisy with effusion, but finally made a complete recovery.

The following illustrates the use of hot water in cases of abortion:—

Case III.—B. D. was attended at her own home. The fœtus came away on January 4th, and was apparently of four months' growth; the placenta was retained, and there was considerable hemorrhage. She came into hospital two days after, and on examination the os was found patulous, and the placenta could be felt through it; all attempts to remove it with the finger, however, failed. There was still a profuse discharge, and the patient appeared weak and anæmic. Hot water was injected, with the effect of immediately stopping the hemorrhage, uterine action set in, and the placenta was expelled five hours after.

*Severe Post-Partum Hemorrhage; Failure of Hot Water; Injection of Liquor Fer. Perchlor. Recovery.*—Case IV.—M. M., aged thirty; second pregnancy. In this case the breech presented, and the funis prolapsed with the escape of the waters; as no pulsation could be felt in it, labor was allowed to proceed, a putrid female child being finally expelled by the natural efforts. The placenta came away in twenty minutes, and the uterus being firmly contracted, the binder was adjusted. Two hours after delivery violent hemorrhage set in; cold napkins were applied to the vulva, friction to the fundus, and ergot injected hypodermically; cold water injected into the uterus, which failing, hot water was injected, and spt. terebinth. ℨss. given by the mouth. As the womb, however, still continued flaccid, the hand was introduced and a quantity of clots removed; at length the uterus contracted, expelling the hand. This favorable result was, however, only of temporary duration, for it again relaxed, and hemorrhage recurred as briskly as ever. The patient was now becoming weak and faint, with a pulse of 185 beats a minute. It was now determined to inject the perchloride of iron. The hand having been again introduced to remove any clots which might have formed in the cavity of the uterus, it was thoroughly washed out with hot water, and about eight ounces of the stypic solution slowly injected. Hemorrhage immediately ceased, and the patient made an excellent recovery.

*Post-Partum Hemorrhage; Injection of Perchloride of iron; Death of Patient.*—Case V.—M. H., aged twenty-three. This woman, who was married to a soldier, had been for some time in bad health attending the out-patient department, but previous to admission into the hospital was so ill that she was unable to leave her bed. When first seen she was very feeble and anæmic, and presented many well-marked symptoms of tertiary syphilis, which disease she said she had acquired two years previously; she also stated that this was her second pregnancy, having previously given birth to an immature and putrid fœtus, and that she feared this child was also dead, as she had not felt its movements for about three weeks. On examination she was found to be already in the second stage of labor, the head presenting. Shortly afterwards the child was born, dead and putrid as predicted. About twenty minutes after this, the placenta not having come away, the pupil in attendance sent for assistance. The uterus was found enormously distended, almost filling the abdomen. On pressure being made, a quantity of blood and clots were expelled with the placenta; cold water was injected into the uterus, but failing to cause contraction it was immediately followed by hot. Ergot and sulphuric ether were at the same time injected hypodermically, after which the womb contracted firmly, but the general symptoms were very alarming—the pulse could not be felt at the wrist, the patient became very restless, endeavoring to sit up, crying out that she would smother, that all was dark around her, she could see nothing. The foot of the bed was immediately raised, and the pillow taken from under her head. At this critical juncture the uterus again relaxed and hemorrhage recurred. The solution of perchloride of iron was produced, but before it could be injected a violent convulsion came on. The case now appeared to be desperate; nevertheless, as a *dernier ressort*, the styptic was resorted to. The tube of a syringe was passed up to the fundus, and about six ounces of the fluid injected. The uterus did not contract; respiration immediately ceased, and she was dead. About forty-five minutes elapsed between the birth of the child and the death of the mother.

*Autopsy.*—There was general thrombosis thoughout the entire venous system; none of the solution had, however, entered the fallopian tubes or the peritoneal cavity.

